# Developing a Sustainable Nutrition Research Agenda in Sub-Saharan Africa—Findings from the SUNRAY Project

**DOI:** 10.1371/journal.pmed.1001593

**Published:** 2014-01-28

**Authors:** Carl Lachat, Eunice Nago, Dominique Roberfroid, Michelle Holdsworth, Karlien Smit, Joyce Kinabo, Wim Pinxten, Annamarie Kruger, Patrick Kolsteren

**Affiliations:** 1 Department of Food Safety and Food Quality, Ghent University, Ghent, Belgium; 2 Unit of Nutrition and Child Health, Institute for Tropical Medicine, Antwerp, Belgium; 3 Department of Nutrition and Food Science, University of Abomey-Calavi, Abomey-Calavi, Benin; 4 Public Health Section, School of Health and Related Research (ScHARR), University of Sheffield, Sheffield, United Kingdom; 5 Africa Unit for Transdisciplinary Health Research, North-West University, Potchefstroom, South Africa; 6 Department of Food Science and Technology, Sokoine University of Agriculture, Morogoro, Tanzania; 7 Faculty of Medicine & Life Sciences, Hasselt University, Diepenbeek, Belgium; 8 Department of Medical Ethics and Philosophy of Medicine, Erasmus MC, Rotterdam, The Netherlands

## Abstract

Patrick Kolsteren and colleagues present the findings of a collaborative effort by stakeholders in sub-Saharan Africa to identify priorities for nutrition research. They propose a new approach that stimulates demand from policy makers for research and holds them accountable for incorporating research into policy and practice.

*Please see later in the article for the Editors' Summary*

**Summary Points**

- Here we present the findings of a collaborative effort by stakeholders in sub-Saharan Africa (SSA) to identify priorities for nutrition research and actions to create an enabling research environment.
- 117 stakeholders from 40 countries in SSA defined priorities using participatory approaches.
- The priority areas for nutrition research were (i) community interventions to improve nutritional status, (ii) behavioral strategies to improve nutritional status, and (iii) food security interventions to improve nutrition.
- The priority actions for creating an enabling nutrition research environment were (i) better governance of nutrition research, (ii) alignment of nutrition research funding with priorities identified within SSA, (iii) increased capacity development for nutrition research competencies, and (iv) enhanced information sharing and communication of nutrition research findings.
- We propose a new approach for nutrition research in SSA that stimulates a demand from SSA policy makers for research in SSA and holds them accountable for incorporating research into policy and practice.

## Nutrition in Sub-Saharan Africa

Despite considerable economic growth in sub-Saharan Africa (SSA) [1], undernutrition rates have not improved compared to other parts of the world [2]. In addition, diet-related noncommunicable diseases have emerged as a public health issue in SSA [3]. Emerging threats, including climatic and demographic changes, affect the nutritional status of populations in SSA and will require effective and innovative mitigation measures [1]. At the same time, there are concerns that scarce resources for actions to improve nutrition are not focused on the interventions with the highest effectiveness [4].

International commitment to address malnutrition has increased, partly because of global food insecurity concerns, academic consensus on effective actions, and the inclusion of nutritional indicators in the Millennium Development Goals [5]. In 2010, the European Commission called for projects to help establish research priorities, strengthen commitment, and identify resource needs, synergies, and coordinated research efforts on a European and global level to tackle malnutrition. The SUNRAY (Sustainable Nutrition Research for Africa in the Years to come) project was selected for funding. SUNRAY took a consultative approach to define priorities for research themes and actions to create an enabling research environment from the perspective of stakeholders in nutrition in SSA. SUNRAY had no a priori focus and considered malnutrition in all its forms and both preventive and curative aspects.

## Methodology

**Figure 1:**
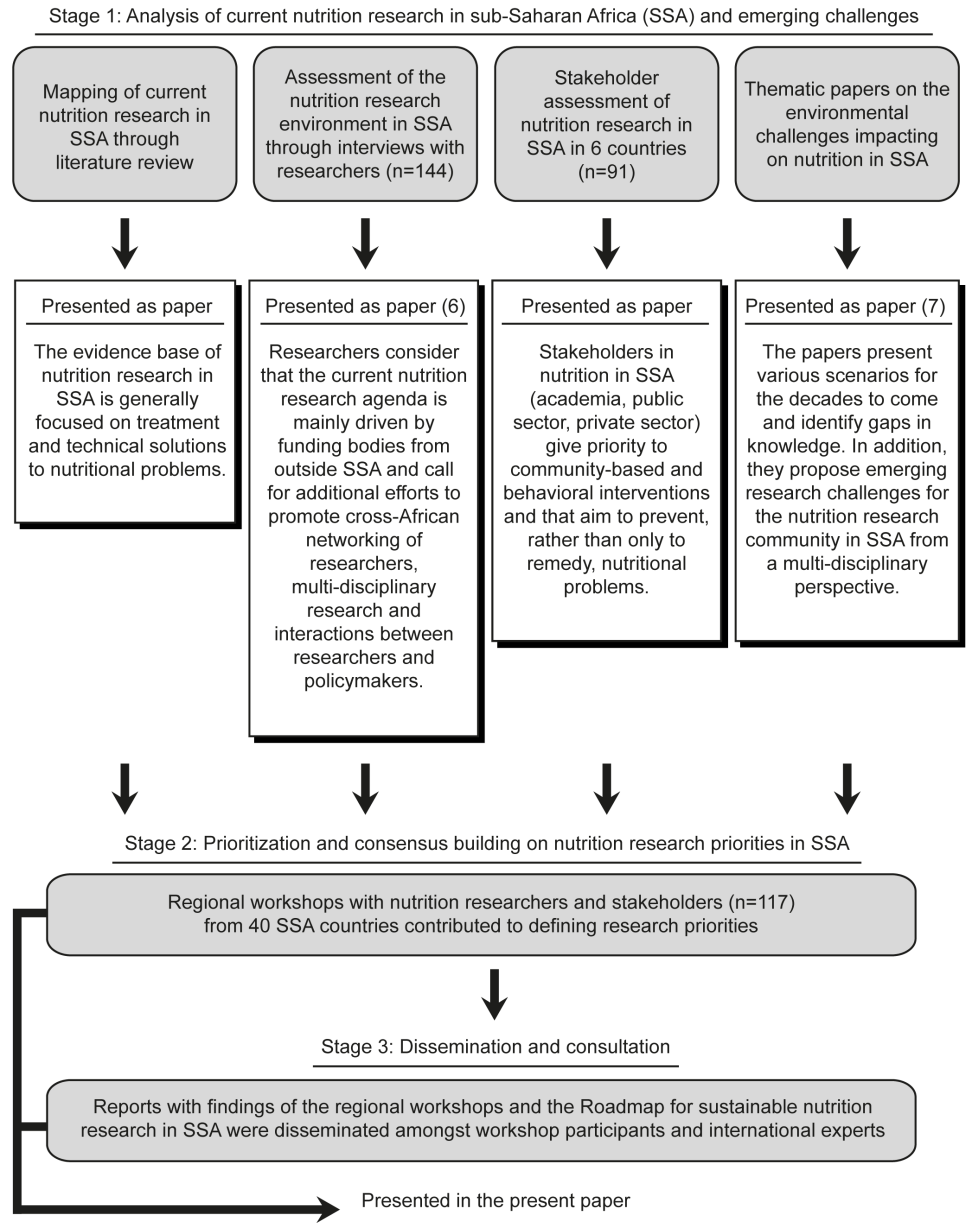
Methodological approach to define priorities and actions for creating an enabling environment for nutrition research in sub-Saharan Africa.

SUNRAY was led by the SUNRAY consortium: academics from four European institutions, academics from four universities in SSA, an international non-governmental organization (NGO), and an organization that funds research in SSA. SUNRAY was organized in three stages (Figure 1). First the SUNRAY consortium analyzed the nutrition research landscape in SSA through a review of institutions publishing nutrition research and type of nutrition research published between 2000 and 2010, an analysis of the perceptions of nutrition researchers regarding nutrition research [6], an assessment of the nutrition research priorities of stakeholders, and the identification of research needs for environmental challenges [7].

In stage 2, three regional workshops in Africa were organized to set priorities. Attendance at these workshops was by invitation only. We circulated invitation letters clarifying the objective of the workshop to researchers and policy makers in SSA selected for their decision-making authority at their institutions. Care was taken to balance the representation of research groups within a country. We aimed for one representative from government and one from academia per country. Selection was primarily based on consultation of three networks—the United Nations (UN) University Food and Nutrition Programme, the International Union of Nutritional Sciences (IUNS), and the Federation of African Nutrition Societies—and those affiliated with dedicated agencies of the UN in the region.

**Figure 2:**
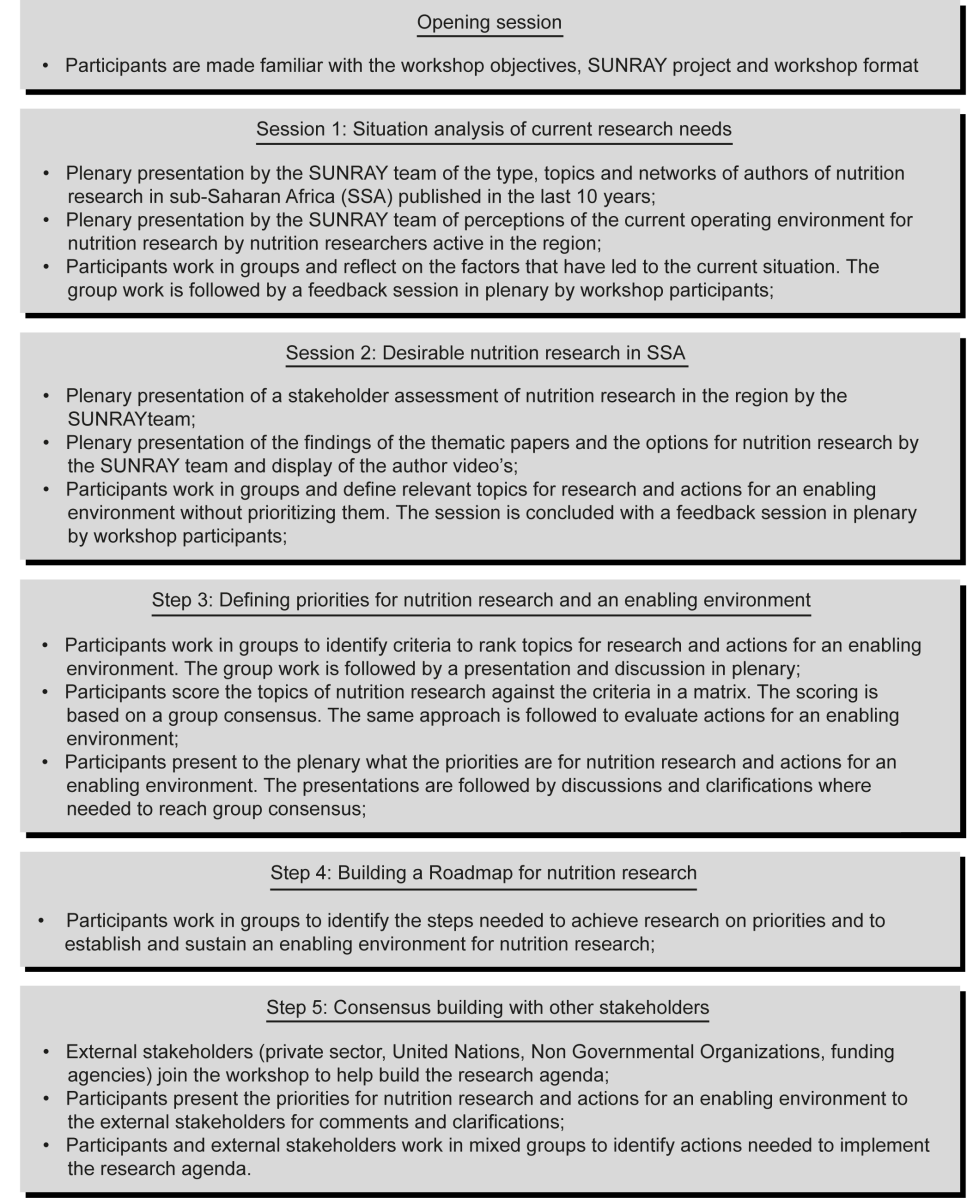
Organization of the regional workshops to define priorities and actions for creating an enabling environment for nutrition research in sub-Saharan Africa.

At the workshops, the findings of stage 1 were presented to set the scene (Figure 2). Next, participants worked in small groups of about eight participants. The groups listed priorities for research and actions for an enabling environment, with criteria to rank them. Next, priorities were ranked by scoring them against the criteria. This approach was used to reach a consensus and allowed a transparent process that considered various criteria [8]. Agreement on the priorities was reached through consensus. Dissenting views were aired and considered within both the working groups as well as the plenary. Each step of the ranking was accompanied by a plenary discussion to enable group understanding and benchmarking. The plenary discussions allowed clarification and consensus building about the emerging priorities. On the last day, external stakeholders (i.e., government officials, UN agencies, NGOs, bilateral donors, and the private sector) were invited to comment on the priorities and helped define the actions needed to implement them (Table S1). No changes to the priorities were made at this point.

The contribution of the SUNRAY consortium was limited to the introductory presentation and feedback on the findings. The moderator essentially facilitated group interactions and free expression.

In stage 3, SUNRAY organized the findings of the regional workshops into priorities for SSA. All priorities emerging from the regional workshop were allocated, and there was no further ranking or analysis afterwards. This report was circulated for consultation and approval by workshop participants from October–November 2012.

Next, SUNRAY prepared a roadmap document with the conclusions and recommendations. During a second consultation round, this document was circulated to the participants and a wider group of 56 international stakeholders, mainly based outside of SSA (two originated from Africa), i.e., academia, multilateral and bilateral donors, the UN, NGOs, IUNS, and representatives of projects in nutrition selected for their excellence or mandate for nutrition in SSA. In addition, face-to-face meetings were organized with the Department for International Development (UK), with the European Commission, and during a national workshop in Benin for Beninese and Togolese stakeholders.

## Results

The results of stage 1 are presented elsewhere [6],. In total, 117 participants from 40 countries in SSA attended the workshops of stage 2 (Table S1); 34 invitees declined to participate, mainly because of prior engagements (*n* = 19). No response was obtained from ten invitees; three invitees provided replacement by colleagues, and two declined but gave no explanation for declining (*n* = 2). Participants were principally senior researchers (52%) and policy makers (30%) in nutrition. The remaining particpants (18%) were external stakeholders.

### Priority Areas for Nutrition Research

The priorities for research that came out of the regional workshops (Table S2) were grouped into three areas (Table 1).

**Table 1:** Priority areas for research and actions for establishing an enabling environment for nutrition research in sub-Saharan Africa, with main objectives and activities needed.

Priorities	Objectives	Activities
**Priority areas for nutrition research**		
Develop effective community-based interventions to improve nutritional status	• To obtain evidence-based knowledge on the sustainable impact of interventions in communities;• To identify the success factors of community-based interventions with regard to the socio-economic and cultural specifics of areas and communities;• To translate evidence-based knowledge and success factors into nutrition-related policies to prevent or address malnutrition.	• Evaluate community-based interventions for their sustainability and effectiveness to reduce and prevent malnutrition including long-term outcomes addressing the development of noncommunicable diseases;• Identify mechanisms for scaling up and sustaining interventions to alleviate malnutrition in communities;• Assess how nutrition research findings can improve policy making or programming to address or prevent malnutrition.
Evaluate behavioral strategies of population groups to improve nutritional status	• To provide the evidence base for policy makers to identify behavioral nutrition interventions for eating and child feeding to prevent malnutrition.	• Take stock of research and the research teams in the area of behavioral nutrition;• Organize a call for a systematic review of the effectiveness of research in SSA on behavior change to improve diet, child feeding, and child caring practices; this will also identify characteristics of unsuccessful programs and specific cultural barriers to improved nutrition and health in vulnerable and neglected groups;• Organize a call for research on interventions to fill the gaps identified from the systematic review of the effectiveness of behavior change interventions, directed to multidisciplinary teams from multiple partners from SSA. A life stage approach should be used to target key population groups for behavior change strategies, e.g., women and children, adolescent girls, urban and rural poor, migrant populations; these strategies should be evaluated using a multidisciplinary approach.
Review food security interventions to improve nutrition	• To provide the evidence base for policy makers to identify food security interventions that effectively mitigate the effect of environmental challenges on nutritional status in SSA.	• Review the potential of (i) microcredit programs for rural development and agriculture; (ii) social protection programs and safety nets; (iii) traditional foods, dishes, and eating habits (composition, utilization, processing) in SSA; and (iv) farming models (i.e., small-scale traditional agriculture versus large-scale modernized systems) to prevent malnutrition in SSA; this review should include a cost-effectiveness analysis and should be conducted from a multidisciplinary perspective;• Develop indicators to study the effect of climate change, water quality, resources, etc., on nutritional outcomes in communities; develop consistent prediction models regarding malnutrition and climate change;• Identify and analyze coping strategies for the most vulnerable populations in SSA to deal with effects of climate change and food insecurity and its effects on nutritional status.
**Priority actions for establishing an enabling environment**		
Ensure better governance for nutrition research	• To ensure better utilization of funding and resources for more action and improved nutritional status of populations.	• Analyze the importance given to research in national development agendas using a multisectoral team and define the national (nutritional) policies addressing nutrition;• Promote good governance of nutrition research and develop a model to manage nutrition research at the national level through a strong multisectoral network;• Set up an advocacy policy for nutrition research findings, including the development of integrated communication plans towards various audiences, i.e., communities, decision makers, NGOs, and funders;• Advocate for better nomination and positioning of nutrition researchers (i.e., at decision-making levels) to favor integration and visibility of nutrition research in policies and increase responsibilities and salaries of nutrition researchers.
Align nutrition research funding with priorities of SSA	• To create opportunities for research groups from SSA to apply for grants, with priority research themes set by stakeholders from SSA, and to create horizontal collaborations so as to increase the research capacity and quality of the studies.	• Organize open calls for research based on transparent and systematically identified research gaps in SSA; this will require the identification of questions (preferably by government agencies), evidence synthesis, and dissemination; priority should be given to research that links different disciplines (agriculture, population, environment, nutrition, etc.) and that focuses on nutritional outcomes while addressing both basic causes and contextual drivers of nutrition;• Establish an African fund for financing multidisciplinary research with a nutrition outcome in SSA.
Increase capacity development for nutrition research	• To build adequate capacity at individual, institutional, and country levels to produce and manage nutrition research	• Establish funding schemes to support local PhD programs, scaling up of sandwich programs and regional mobility for MSc training in SSA, and refresher courses for various competencies, e.g., good research practice and scientific leadership;• Map the research and training efforts in SSA so that research gaps can be addressed effectively, networking can surmount language barriers, and possibilities for short (regional) training programs are clear;• Develop networks in SSA that focus on future challenges linking climatic change, socio-demographic trends, and water issues with nutrition.
Enhance information sharing and communication of nutrition research findings	• To facilitate uptake of up-to-date and scientifically sound nutrition research findings and the effectiveness of nutrition-sensitive interventions;• To establish a dialogue between the nutrition research community, policy makers, and the community to facilitate use of findings from local nutrition research.	• Establish a hub to centralize, screen, and index findings from nutrition research relevant for SSA; non-academic literature would require an assessment and peer review of its scientific rigor;• Create training opportunities for nutrition researchers to develop skills for effective communication with policy makers, the community, and other stakeholders.

#### Evaluate the impact of community interventions

Participants emphasized that interventions for malnutrition have focused on curative aspects. Community-based initiatives that create an environment to prevent malnutrition using locally available approaches and resources, i.e., nutrition-sensitive approaches from areas such as agriculture, education, family planning, environmental sanitation, and rural development should be evaluated for their effectiveness.

#### Effectiveness of behavioral strategies

Although some approaches have been tested in SSA, participants suggested that more understanding of the drivers of eating and child feeding behavior in SSA is needed to design effective interventions. Such research would require the propagation of multidisciplinary research across the continent that includes disciplines such as psychology and the social sciences.

#### Exploit the potential of food security interventions

Sustainability and the potential to mitigate the effect of environmental challenges on nutritional status should be assessed for social safety nets, e.g., conditional/unconditional cash transfers (see [9]), and for food security interventions, e.g., the promotion of traditional foods, food systems, and local adaptation and mitigation strategies for environmental challenges.

### Priority Actions for Creating an Enabling Research Environment

The priority actions for creating an enabling nutrition research environment were organized into four actions for SSA (Table S3).

#### Better governance of nutrition research

Better governance and uptake of nutrition research is needed to ensure the practical relevance of findings, so that efforts can be targeted towards the priorities for action. A clear integration of nutrition research in the development agenda, with an explicit articulation of priorities for nutrition research in policies in SSA was highlighted.

#### Alignment of nutrition research funding with priorities in SSA

The current nutrition research agenda was perceived to be driven by high-income countries, while the capacity to attract international competitive funding for most researchers in SSA was considered limited. There is a need to align funding for nutrition in SSA with the priorities identified by stakeholders from SSA.

#### Increased capacity development for nutrition research

Efforts to increase regional networking and mobility within SSA, re-entry grants for promising scientists, and various specific courses are needed to capitalize on the existing nutrition research capacity in SSA. Scholarship programs from donor countries might need revision to favor local PhD programs and exchange programs where students carry out research at universities within SSA.

#### Enhanced information sharing and communication of research

A new approach is needed to rationalize the communication of nutrition research findings with relevance for SSA. Mass-distributed reports or guidelines from various stakeholders in nutrition in SSA need to be organized systematically, with a transparent appraisal of quality. Local research findings should be communicated effectively to decision makers in SSA.

Regarding stage 3, 39 workshop participants commented on the report and endorsed it. A response rate of 27% (15/56) for international stakeholders was obtained. The feedback received was overall positive and highlighted the global significance of the SUNRAY findings.

## Discussion

Our priorities for nutrition research focus on the prevention of malnutrition in all its forms and deviate substantially from previous initiatives [10],[11] that listed micronutrient supplementation or fortification as top priorities for research or investment. The participants expressed concerns regarding the sustainability of technological and curative approaches and prioritized research to prevent malnutrition. Most of the identified priorities called for multidisciplinary research and corresponded with those of a global nutrition research agenda [12].

Our findings reiterate previous concerns and illustrate that the prioritizing of nutrition research topics must be accompanied by a better environment and translation of research into action [5],[13],[14]. Recent research priority-setting exercises for SSA [12],[15]–[18] emphasize that motivating and educating policy makers in SSA is critical to improve health [15]. A critical challenge for scaling up efforts for nutrition in SSA is integrating nutrition research findings in programs and policies. Our findings illustrate the need to integrate different levels of nutrition research. They respond to an earlier call for new frameworks for the production and use of nutrition knowledge to enhance its practical utility for stakeholders and societal benefit [19].

Systematic reviews have gained ground in nutrition [20],[21]. A renewed approach for nutrition research needs to foster specific mechanisms to translate this evidence into context-specific recommendations for decision makers in SSA. Similar to health research [22], developing a nutrition research agenda should be an inclusive process initiated by decision makers in SSA in collaboration with other stakeholders. Much like health technology assessment (HTA), such a process should follow transparent and well-established procedures to ensure an objective outcome. Experiences with HTA are limited in SSA [23].

An important condition for the success of HTA for nutrition is the presence of a strong research community. In many countries of SSA the nutrition research community is weak and fragmented [4], and adequate capacity to govern nutrition research [5] together with policy commitment and funding is required.

Following SUNRAY, we have piloted an annual course on evidence-based nutrition and initiated the development of a knowledge network for evidence-based nutrition in Africa. This network will focus on the use and adaptation of existing evidence in policy and programming in Africa and on developing appropriate tools for decision makers. Facilitating evidence-based decisions is expected to improve the effectiveness of nutrition actions in SSA. Under its African presidency, the IUNS requires support, together with the Federation of African Nutrition Societies and national nutrition societies, to drive the development of a revised approach to nutrition research in SSA. The knowledge network will be able to support this process.

Setting a nutrition research agenda is also a normative process. SUNRAY went beyond international research guidelines [24], as it involved the integration of values and concerns of stakeholders in SSA. As such, our process also aligned with the principles of the Busan declaration [25].

A review of priority setting exercises in low- and middle-income countries illustrates the current lack of both systematic approaches and the involvement of stakeholders [26]. SUNRAY successfully provided a forum for stakeholders from SSA, including countries where nutrition research is poorly developed. Previous exercises for priority setting for research in low- and middle-income countries [11],[12],[15]–[17],[27] did not focus on creating momentum within the research community or on enhancing the research environment. We acknowledge a large heterogeneity of participants. Not all participants were able to make equally informed decisions on priorities across all issues. Another limitation was the low response rate of the consultation round with international stakeholders, which we attribute to the short duration of the consultation round and the high-level profile of those contacted. Clearly, the relative importance of our findings differs by region and country. Although the prioritization process was based on a comprehensive assessment of literature and a broad-based consultation process in SSA, we acknowledge a potential selection bias. We limited workshop participants per country to enable mutual dialogue and interactions in smaller groups.

International commitments such as Scaling Up Nutrition will require actions at the country level to yield success on the ground. A crucial condition for this success is to build and strengthen national research capacity that can engage effectively with policy makers. Our findings call for a nutrition research system that stimulates a demand from policy makers from SSA for research in SSA and holds them accountable for incorporating research into policy.

## Supporting Information

Table S1 **Participants of the regional workshops.**
(DOCX)

Table S2 **Ranking criteria and priorities for nutrition research in sub-Saharan Africa, organized by regional workshop.**
(DOCX)

Table S3 **Ranking criteria and priority actions for establishing an enabling environment for nutrition research in sub-Saharan Africa, organized by regional workshop.**
(DOCX)

## Abbreviations

HTA: health technology assessment
IUNS: International Union of Nutritional Sciences
NGO: non-governmental organization
SSA: sub-Saharan Africa
UN: United Nations

## References

[1] United Nations Development Programme (2012) Africa human development report: towards a food secure future. Washington (District of Columbia): United Nations Development Programme.

[2] Stevens GA, Finucane MM, Paciorek CJ, Flaxman SR, White RA, et al (2012) Trends in mild, moderate, and severe stunting and underweight, and progress towards MDG 1 in 141 developing countries: a systematic analysis of population representative data. Lancet 380: 824–834. 22770478 10.1016/S0140-6736(12)60647-3 PMC3443900

[3] Kelly T, Yang W, Chen CS, Reynolds K, He J (2008) Global burden of obesity in 2005 and projections to 2030. Int J Obes (Lond) 32: 1431–1437. 18607383 10.1038/ijo.2008.102

[4] Morris SS, Cogill B, Uauy R (2008) Effective international action against undernutrition: why has it proven so difficult and what can be done to accelerate progress? Lancet 371: 608–621 doi:10.1016/S0140-6736(07)61695-X. 18206225 10.1016/S0140-6736(07)61695-X

[5] Gillespie S, Haddad L, Mannar V, Menon P, Nisbett N (2013) The politics of reducing malnutrition: building commitment and accelerating progress. Lancet 382: 552–569 doi:10.1016/S0140-6736(13)60842-9. 23746781 10.1016/S0140-6736(13)60842-9

[6] van Royen K, Lachat C, Holdsworth M, Smit K, Kinabo J, et al (2013) How can the operating environment for nutrition research be improved in sub-Saharan Africa? The views of African researchers. PLoS ONE 8: e66355 doi:10.1371/journal.pone.0066355. 23776663 10.1371/journal.pone.0066355 PMC3680459

[7] SUNRAY (2012) Challenges for nutrition in sub-Saharan Africa: background documents for the SUNRAY regional workshops. Available: http://sunrayafrica.co.za/sunray_cms/downloads/dynamic/compound_text_content/sunray_background_papers_english_0d1fe7c4b9d1c53e38a94f6ea689eb07.pdf. Accessed 19 December 2013.

[8] Baltussen R, Niessen L (2006) Priority setting of health interventions: the need for multi-criteria decision analysis. Cost Eff Resour Alloc 4: 14 doi:10.1186/1478-7547-4-14. 16923181 10.1186/1478-7547-4-14 PMC1560167

[9] Ruel MT, Alderman H (2013) Nutrition-sensitive interventions and programmes: how can they help to accelerate progress in improving maternal and child nutrition? Lancet 382: 536–551 doi:10.1016/S0140-6736(13)60843-0. 23746780 10.1016/S0140-6736(13)60843-0

[10] Copenhagen Consensus 2012 (2012) Nobel Laureates: more should be spent on hunger, health. Available: http://www.copenhagenconsensus.com/sites/default/files/CC12%2BResults%2BPress%2BRelease%2BFinal_0.pdf. Accessed 19 December 2013.

[11] Brown KH, Hess SY, Boy E, Gibson RS, Horton S, et al (2009) Setting priorities for zinc-related health research to reduce children's disease burden worldwide: an application of the Child Health and Nutrition Research Initiative's research priority-setting method. Public Health Nutr 12: 389–396 doi:10.1017/S1368980008002188. 18426636 10.1017/S1368980008002188

[12] The Sackler Institute for Nutrition Science (2013) A global agenda for nutrition science. Outcome of a collaborative process between academic and non-profit researchers and the World Health Organization. Available: http://www.nutritionresearchagenda.org/pdf/Sackler-Agenda-121313-WEB.pdf. Accessed 19 December 2013.

[13] Berg A (1993) Sliding toward nutrition malpractice: time to reconsider and redeploy. Am J Clin Nutr 57: 3–7. 8257461 10.1093/ajcn/57.1.3

[14] Berg A, Levinson F, Moorthy D (2008) Reflections from the front lines: swimming upstream with optimism. SCN News 36: 44–50 Available: http://www.unscn.org/layout/modules/resources/files/scnnews36.pdf. Accessed 19 December 2013.

[15] Rudan I, Kapiriri L, Tomlinson M, Balliet M, Cohen B, et al (2010) Evidence-based priority setting for health care and research: tools to support policy in maternal, neonatal, and child health in Africa. PLoS Med 7: e1000308 doi:10.1371/journal.pmed.1000308. 20644640 10.1371/journal.pmed.1000308 PMC2903581

[16] Swingler GH, Irlam JH, Macharia WM, Tietche F, Meremikwu MM (2005) A systematic review of existing national priorities for child health research in sub-Saharan Africa. Health Res Policy Syst 3: 7 doi:10.1186/1478-4505-3-7. 16300672 10.1186/1478-4505-3-7 PMC1315320

[17] Tomlinson M, Chopra M, Sanders D, Bradshaw D, Hendricks M, et al (2007) Setting priorities in child health research investments for South Africa. PLoS Med 4: 1293–1298. 10.1371/journal.pmed.0040259 PMC1952202 17760497

[18] Wazny K, Zipursky A, Black R, Curtis V, Duggan C, et al (2013) Setting research priorities to reduce mortality and morbidity of childhood diarrhoeal disease in the next 15 years. PLoS Med 10: e1001446 doi:10.1371/journal.pmed.1001446. 23690756 10.1371/journal.pmed.1001446 PMC3653794

[19] Pelletier DL, Porter CM, Aarons GA, Wuehler SE, Neufeld LM (2013) Expanding the frontiers of population nutrition research: new questions, new methods, and new approaches. Adv Nutr 4: 92–114 doi:10.3945/an.112.003160. 23319128 10.3945/an.112.003160 PMC3648745

[20] Bhutta ZA, Ahmed T, Black RE, Cousens S, Dewey K, et al (2008) What works? Interventions for maternal and child undernutrition and survival. Lancet 371: 417–440. 18206226 10.1016/S0140-6736(07)61693-6

[21] World Health Organization (2013) E-library of Evidence for Nutrition Actions (eLENA). Available: http://www.who.int/elena/en/ [database]. Accessed 19 December 2013.

[22] Mckee M, Stuckler D, Basu S (2012) Where there is no health research: what can be done to fill the global gaps in health research? PLoS Med 9: e1001209. 22545025 10.1371/journal.pmed.1001209 PMC3335864

[23] International Network of Agencies for Health Technology Assessment (2012) Members. Available: http://www.inahta.org/Members/. Accessed 19 December 2013.

[24] World Medical Association Declaration of Helsinki: ethical principles for medical research involving human subjects. JAMA 310: 2191–2194 doi:10.1001/jama.2013.281053. 10.1001/jama.2013.281053 24141714

[25] (2011) Busan partnership for effective development co-operation. 4th High Level Forum on Aid Effectiveness; 29 Nov–1 Dec 2011; Busan, Korea. Available: http://effectivecooperation.org/files/OUTCOME_DOCUMENT_-_FINAL_EN.pdf. Accessed 19 December 2013.

[26] Tomlinson M, Chopra M, Hoosain N, Rudan I (2011) A review of selected research priority setting processes at national level in low and middle income countries: towards fair and legitimate priority setting. Health Res Policy Syst 9: 19 doi:10.1186/1478-4505-9-19. 21575144 10.1186/1478-4505-9-19 PMC3115910

[27] Costello A, Filippi V, Kubba T, Horton R (2007) Research challenges to improve maternal and child survival. Lancet 369: 1240–1243 doi:10.1016/S0140-6736(07)60574-1. 17434384 10.1016/S0140-6736(07)60574-1

