# Supplementary material for: Developing a Sustainable Nutrition Research Agenda in Sub-Saharan Africa—Findings from the SUNRAY Project
Source: PLoS Med. 2014 Jan 28;11(1):e1001593. doi: 10.1371/journal.pmed.1001593 (PMC3904839; doi:10.1371/journal.pmed.1001593)
Supplement: Table S1 — Participants of the regional workshops. (DOCX) [file pmed.1001593.s001.docx]

**Supporting information Table S1** Participants of the regional workshops

|  | Tanzania | Benin | South Africa |
| --- | --- | --- | --- |
| Academia | - Ethiopia: Hawassa University - Ghana: Regional Institute for Population Studies, University of Ghana, Millennium village project; Noguchi memorial institute for medical research & University of Ghana - Kenya: University of Nairobi & Kenyatta University - Malawi: Department of nutrition HIV & AIDS - Nigeria: Department of Human Nutrition, Faculty of Public Health, University of Ibadan - Tanzania: Sokoine University of Agriculture; Open University of Tanzania - The Gambia: MRC International Nutrition Group - Uganda: Makarere University, School of Public Health & Uganda Action for Nutrition - Zambia: University of Zambia | - Benin: University of Abomey-Calavi - Burkina-Faso: University of Ouagadougou; Institute for Research and Applied Sciences and Technologies - Burundi: University of Bujumbura - Cameroun: Department of Biochemistry, Center for Food Research and Nutrition, IMPM/MINRES & University of Douala - Central African Republic: University of Bangui - Chad: Chadian Institute for Agricultural Research and Development - Cote d’Ivoire: University of Cocody - Republic of Guinea: University of Conakry & National Institute for Public Health - Madagascar: University of Majunga - Mali: Nutrition Department, National Institute for Research in Public Health - Niger: University of Adbou Moumouni - Senegal: Laboratory of Nutrition, Faculty of Sciences et Technology, University of Cheikh Anta Diop - Togo: University of Lomé | - Botswana: University of Botswana & Princess Marina Hospital and for Government - Cape Verde: University of Cape Verde - Ghana: University of Ghana - Lesotho: National University of Lesotho - Mozambique: Eduardo Mondlane University - South Africa: North-West University, Stellenbosch University, University of Pretoria & University of the Free State - Zimbabwe: University of Zimbabwe |
| Government | - Liberia: Ministry of Agriculture, Ministry of Health and Social Welfare - Malawi: Department of Nutrition & HIV; Office of Presidential Labor/ANHA - Nigeria: Federal Ministry of Health, Family Health Department Nutrition Division - Rwanda: Ministry of Health - Seychelles: Public Health Department, Ministry of Health - South Sudan: State Ministry of Agriculture and Forestry - Tanzania: Food and Nutrition Centre; Ministry of Agriculture Food Security & Cooperatives - The Gambia: National Nutrition Agency - Uganda: Ministry of Health - Zambia: National Food and Nutrition Commission | - Benin: President of the National Assembly; Directorate of Family Health, Ministry of Public Health - Burkina-Faso: Directorate of Nutrition, Ministry of Health - Burundi: Nutrition Unit, Ministry of Health - Central African Republic: Ministry of Health - Chad: National Centre for Nutrition and Food Technology, Ministry of Health - Ivory Coast: Directorate of Research and Health; Service of food and nutrition, Ministry of health and Environmental Sanitation - Republic of Guinea: National Nutrition Program, Ministry of Public Health - Madagascar: Office National de Nutrition - DR Congo: National Nutrition Program - Republic of the Congo: Ministry of Health - Togo: National Service of Nutrition, Ministry of Health | - Guinea Bissau: Ministry of Health - Lesotho: Dietetics Department at the Ministry of Health and Social Welfare - Mozambique: Ministry of Health & Technical Secretariat for Food and Nutrition Security at the Ministry of Agriculture - Sao Tomé: Ministry of Health - Swaziland: Swaziland National Nutrition Council & Ministry of Health - Zimbabwe: Ministry of Health and Child Welfare |
| Stakeholders that participated on day 3 | - Kenya: FHI 360 - Rwanda: FHI 360 & Rwanda Nutrition Society - Tanzania: World Food Program, Helen Keller International, Tanzania consortium of nutritionists; Said Salim Bakhresa & Co Ltd, Tanzania Food & Drug Authority; The Centre for Counseling, Nutrition and Health Care; USAID - Uganda: World Food Program, Regional office - Zambia: Nutrition Association of Zambia | - Benin: Belgian Embassy, European Commission - France: Action Against Hunger - Senegal: UNICEF Regional office | - South Africa: South African Nutrition Society; Oxfam; Global Alliance for Improved Nutrition; South African Sugar Association; Private Food and Safety Consultant - Swaziland: Swaziland Infant Nutrition Action Network |
