# Supplementary material for: Developing a Sustainable Nutrition Research Agenda in Sub-Saharan Africa—Findings from the SUNRAY Project
Source: PLoS Med. 2014 Jan 28;11(1):e1001593. doi: 10.1371/journal.pmed.1001593 (PMC3904839; doi:10.1371/journal.pmed.1001593)
Supplement: Table S2 — Ranking criteria and priorities for nutrition research in sub-Saharan Africa, organized by regional workshop. (DOCX) [file pmed.1001593.s002.docx]

**Supporting information Table S2** Ranking criteria and priorities for nutrition research in sub-Saharan Africa organized by regional workshop

| Tanzania | Benin | South Africa |
| --- | --- | --- |
| Ranking criteria | | |
| - Impact on nutritional status - Community relevance - Feasibility - Added value of new evidence - National and regional priority - Innovation and novelty | - Relevance - Impact - Cost - Effectiveness - Feasibility | - Impact - Relevance - Feasibility - Capacity - Multisectoral |
| Priorities for nutrition research in SSA | | |
| - Intervention research geared towards prevention of malnutrition - Develop small scale farming models which are resilient to economic and climate challenges in securing food and nutrition security - Research that relates demography and food or nutrition security - Review the effectiveness of health and nutrition interventions in SSA | - Research on the functional properties and optimal use of local foods from SSA with evaluation of their impact on nutrition status and the prevention of malnutrition - Research on food crops that are resistant to climatic changes - Research on effective post-harvest technologies to improve - Research on effective mitigation strategies for price volatility and nutritional status of vulnerable consumers - Impact of communication strategies on dietary habits of populations. - Research on specific diets in SSA and their influence on nutrition status - Effects of land grabbing (e.g. biofuels) and the nutritional status of those affected - Evaluation of social dynamics, demographic transition, epidemiological transition and nutritional transition to prevent malnutrition - Research to develop strategies for countries with water scarcity to ensure sufficient agricultural production - Effectiveness of microcredit for better food security - Improvement of animal nutrition to improve human nutrition | - Evaluation of the implementation of high impact nutrition interventions and nutrition sensitive interventions across SSA; Influence strategies to implement and scaling up high impact nutrition interventions and nutrition sensitive interventions in countries - Impact of climate change and social dynamic factors on nutrition and food security; What are the best predictive models and indicators for future impact of climate change on Nutrition; (resilience, stunting, migration); Water management model to improve agriculture, health, nutrition and food security. Research on more effective food production, preservation, storage, process, access and utilization at all levels (including indigenous and traditional foods) - Evaluate different models of behavior change related to nutrition; Determine strategies to overcome community specific cultural barriers to improved nutrition and health of vulnerable and neglected groups - Evaluation of government policies that have impact on nutrition and food security; Investigate effective translation of local research findings into policies and frameworks - Investigate interventions for the prevention & management of non-communicable diseases across the life cycles in SSA. - Identify innovative strategies for prevention & management of nutrition-related problems of communicable diseases |
