# Supplementary material for: Developing a Sustainable Nutrition Research Agenda in Sub-Saharan Africa—Findings from the SUNRAY Project
Source: PLoS Med. 2014 Jan 28;11(1):e1001593. doi: 10.1371/journal.pmed.1001593 (PMC3904839; doi:10.1371/journal.pmed.1001593)
Supplement: Table S3 — Ranking criteria and priority actions for establishing an enabling environment for nutrition research in sub-Saharan Africa, organized by regional workshop. (DOCX) [file pmed.1001593.s003.docx]

**Supporting information Table S3** Ranking criteria and priority actions for an enabling environment for nutrition research in sub-Saharan Africa organized by regional workshop

| Tanzania | Benin | South Africa |
| --- | --- | --- |
| Ranking criteria | | |
| - Relevance - Motivation of researchers - Inclusive participation - Effectiveness - Feasibility - Sustainability | - Relevance - Feasibility - Cost-effectiveness - Impact - Sustainability | - Reach and impact - Sustainability - Do-able - Resources - Support |
| Priority actions for an enabling environment for nutrition research in SSA | | |
| - Funding is critical for effective nutrition research - Identification of dimensions of capacity for nutrition research - Render nutrition research demand driven to ensure better uptake, utilization and effectiveness of decision-making. This arises from inclusive participation - Provide the capacity (e.g., knowledge base) to the communities to demand nutritional entitlements through their leaders (specifically, parliament representatives). In turn this will prompt demand for nutrition research from the parliaments. | - Creation of a fund for research on nutrition in SSA. Financing needs to be aligned with priority research themes from SSA - Improvement of research capacity of researchers and their institutions. Training of researchers, improvement of existing infrastructure - Multisectoral collaboration, knowledge and experience sharing and creation of a network. Mobilization of researchers, consultation in decision making and partnership with stakeholders - Integration of research in nutrition in regional, national and international policies; development of a strategic research plan - Improved access to information and communication: Support for researcher to access the Internet, scientific databases and publications. - Good governance i.e. better project management | - Dissemination of research results (publications, networking, evidence-based advocacy/lobbing, workshops, organization of nutrition summer schools for scientists, attending scientific conferences, reports) - Scientific leadership and mentorship (develop competence as top-rated scientist, develop ability to think and plan ahead, develop competence to transform plans to action, develop project, management skills, develop creative problem solving skills, develop ability to build effective teams, develop a culture to develop others, develop value driven way of working (honesty, integrity, trustworthiness etc.), create opportunities for younger scientists) - Tertiary nutrition training (assess the nutrition needs per country, develop postgraduate programs using expertise from universities in SSA or abroad) - Infrastructure |
